# Generation of monoclonal pan-hemagglutinin antibodies for the quantification of multiple strains of influenza

**DOI:** 10.1371/journal.pone.0180314

**Published:** 2017-06-29

**Authors:** Aziza P. Manceur, Wei Zou, Anne Marcil, Eric Paquet, Christine Gadoury, Bozena Jaentschke, Xuguang Li, Emma Petiot, Yves Durocher, Jason Baardsnes, Manuel Rosa-Calatrava, Sven Ansorge, Amine A. Kamen

**Affiliations:** 1 Human Health Therapeutics, National Research Council Canada, Montréal, Québec, Canada; 2 Centre for Biologics Evaluation, Biologics and Genetic Therapies Directorate, Health Canada, Ottawa, Ontario, Canada; 3 Laboratoire de Virologie et Pathologie Humaine—VirPath Team, International Center for Infectious diseases Research, Inserm U1111, CNRS UMR5308, ENS Lyon, Université Claude Bernard Lyon 1, Villeurbanne, France; 4 Department of Bioengineering, McGill University, Montréal, Québec, Canada; University of South Dakota, UNITED STATES

## Abstract

Vaccination is the most effective course of action to prevent influenza. About 150 million doses of influenza vaccines were distributed for the 2015–2016 season in the USA alone according to the Centers for Disease Control and Prevention. Vaccine dosage is calculated based on the concentration of hemagglutinin (HA), the main surface glycoprotein expressed by influenza which varies from strain to strain. Therefore yearly-updated strain-specific antibodies and calibrating antigens are required. Preparing these quantification reagents can take up to three months and significantly slows down the release of new vaccine lots. Therefore, to circumvent the need for strain-specific sera, two anti-HA monoclonal antibodies (mAbs) against a highly conserved sequence have been produced by immunizing mice with a novel peptide-conjugate. Immunoblots demonstrate that 40 strains of influenza encompassing HA subtypes H1 to H13, as well as B strains from the Yamagata and Victoria lineage were detected when the two mAbs are combined to from a pan-HA mAb cocktail. Quantification using this pan-HA mAbs cocktail was achieved in a dot blot assay and results correlated with concentrations measured in a hemagglutination assay with a coefficient of correlation of 0.80. A competitive ELISA was also optimised with purified viral-like particles. Regardless of the quantification method used, pan-HA antibodies can be employed to accelerate process development when strain-specific antibodies are not available, and represent a valuable tool in case of pandemics. These antibodies were also expressed in CHO cells to facilitate large-scale production using bioreactor technologies which might be required to meet industrial needs for quantification reagents. Finally, a simulation model was created to predict the binding affinity of the two anti-HA antibodies to the amino acids composing the highly conserved epitope; different probabilities of interaction between a given amino acid and the antibodies might explain the affinity of each antibody against different influenza strains.

## Introduction

Influenza is a contagious disease that can lead to hospitalization, and even death for vulnerable patients. The influenza virus belongs to the *Orthomyxoviridae* family and is classified as type A, B or C. Annual influenza infections are caused by type A, and to a lesser extent by type B [1]. Two viral surface glycoproteins are used to identify the subtype: hemagglutinin (HA) and neuraminidase (NA). Type A can further be classified into two phylogenetic groups based on HA (group 1 and 2). Disease prevention is achieved through vaccination, and the production of vaccine lots is initiated several months before the flu season based on predictions. When vaccines are well matched with the circulating strains, vaccination offers up to 83% protection [2]. Unfortunately, new strains of influenza emerge every few years and lead to a mismatch between the predicted vaccine strains and the circulating strains, which can significantly decrease the efficacy of the vaccine. Currently, sixty percent of influenza vaccines are still produced by inoculating fertilized eggs, but new production platforms have been reported or are being used such as insect-cell cultures [3], other mammalian cell lines [4–9] as well as plant-based vaccines [10–13].

Despite the progress made in the influenza vaccine industry, the quantification remains a challenge. The main quantification method approved by regulatory agencies is the single radial immunodiffusion (SRID) assay, which is used to quantify HA. The dose of a trivalent or quadrivalent influenza vaccine is typically 45μg to 60 μg HA (15μg of each strain). The SRID assay has been used by the influenza vaccine community since 1978 and a large amount of historical data is available [14]. The main limitation however, is that it necessitates the use of strain-specific anti-HA polyclonal antibodies and calibrating-antigens. The production of SRID reagents requires about three months which can significantly slow down the release of new vaccines, and constitutes a major limiting factor in the case of pandemics. The two main options would therefore be to generate universal SRID-reagents that could be used with all influenza strains, or to adopt an alternative quantification method that would not be limited by the availability of reagents. A number of putative quantification methods were reviewed recently [15].

To support the progress of alternative quantification methods and to speed up process development of new vaccines, antibodies that can recognize HA from multiple strains of influenza are a valuable tool. For instance, a polyclonal antibody named Uni-1 was generated in rabbits against a highly conserved sequence found between the two subunits of HA (the fusion peptide) [16]. Thirteen influenza A subtypes, as well as an influenza B subtype were detected by Uni-1. However, the yield of antibody was limited to what could be produced in a rabbit. Similarly to other polyclonal antibodies, Uni-1 also suffers from lot-to-lot variations which leads to heterogeneity in specificity and binding affinity.

The goal of this work is therefore to develop high-quality monoclonal pan-HA antibodies to ensure a constant production system and to respond to industrial needs. Immunoblotting data demonstrates that 40 strains of influenza belonging to 13 HA subtypes are detected by a cocktail of two antibodies named F211-10A9 and F211-11H12. Furthermore, the antibodies can be used in a dot blot assay and a competitive ELISA for quantification purposes. After sequencing the two selected pan-HA antibodies, plasmids were generated and transfected in CHO cells. The binding affinity and specificity of antibodies produced in CHO pools is similar to the ones of antibodies produced from mouse hybridomas, paving the path for large scale production. Finally, a model was built to help us understand the binding of the pan-HA antibodies to the highly conserved peptide sequence used to raise an immune response.

## Material and methods

### Peptide-conjugate synthesis

Immunogen consists of a peptide sequence corresponding to the fusion peptide at the N-terminal of HA2 (GLFGAIAGFIEGGW) which is highly conserved among influenza strains and subtypes. The peptide was modified to make it more soluble and the Thio-Cys at C-terminus of the functionalized antigen was conjugated to Bromoacetylated Keyhole Limpet Hemocyanin (KLH). To perform bromoacetylation of KLH, 20 mg of KLH (Sigma-Aldrich) was reconstructed by the addition of 2 mL of deionized water at room temperature, and exchanged to 10 x PBS by an Amicon Ultra centrifugal filter (MWC 30K). To the above solution of KLH in 10 X PBS (2 mL) was added 9 mg of bromoacetic acid N-hydroxysuccinimide ester in DMSO (0.18 mL) at 4°C overnight. The product was purified by a G-25 column (50 x 1.6 cm) with PBS as eluent and bromoacetyl KLH obtained was stored in PBS buffer. Similarly, bromoacetylated BSA was also made and MALDI indicated 9–10 bromoacetyl groups per BSA; we expect similar ratio of bomoacetyl group present in KLH. The peptide conjugates were prepared via a thio-ether bond between terminal Cys of the peptide antigen and bromoacetylated KLH under the conditions of 0.1M phosphate buffer with 5 mM EDTA-0.01% sosium azide at pH 8.0–8.5 overnight at room temperature. As a reference to estimate the peptide antigen on KLH, the bromoacetylated BSA described above was also coupled with the same peptide (GLFGAIAGFIEGGW) to give a conjugate with a ratio of peptide:BSA 6–7:1. The peptide antigen on KLH was presumed having a similar w/w ratio as BSA.

### Antibody production from mouse hybridomas

Six-week-old female A/J mice (The Jackson Laboratory, Bar Harbor,ME) were used. This study was carried out in strict accordance with Canadian Council on Animal Care policy and guidelines. Animal protocol was reviewed and approved by the Animal Care Committee of Biotechnology Research Institute at National Research Council of Canada (protocol #13-MAR-I-061). All efforts were made to minimize suffering. Spleen was removed following euthanasia. Anesthesia was achieved by inhalation using isoflurane at 4–5% (induction) and maintaining at 2%. Euthanasia was performed by exsanguination under anesthesia, followed by cervical dislocation. Mice were immunized intraperitoneally and subcutaneously with 100 μg of peptide-conjugates emulsified in Titermax adjuvant (Cedarlane, Burlington, Ont) and boosted at day 22. A final boost was done at day 80 with 100 μg of conjugate in PBS four days prior to fusion. Hybridoma generation was done as described previously [17]. Hybridoma supernatants were screened for appropriate antigen specificity by enzyme-linked immunosorbent assay (ELISA) on biotinylated peptide. The reagent was prepared by mixing the peptide antigen with Nε-NH2-PEG2-propionamide-Lys (2.5 mg) with biotin N-hydroxysuccinimide ester (0.5 mg, Sigma-Aldrich, H1759) in DMSO (0.5 mL) at room temperature. The solution was kept for 5 hours, followed by dilution with water and lyophilisation. The product was characterized by MALDI and no further purification was needed for ELISA.

Briefly, half-area 96-well plate were coated with 25 μl per well of NeutrAvidin (Thermofisher, Rockford, IL) in 50 mM carbonate buffer at pH 9.8. After 2-hour incubation at room temperature, microplates were washed three times in PBS and blocked for 30 min with 1% bovine serum albumin (BSA). Microplates were washed once with PBS and 25 μl of biotinylated peptide at 5μg/ml was added and incubated overnight at 4°C. After 4 washes with PBS containing 0.05% Tween 20 (PBS-Tween), 25 μl of monoclonal antibody supernatant was added. After a 2-h incubation at 37°C, 5% CO2, microplates were washed 3 times with PBS-Tween and 25 μl of a 1/5,000 dilution of alkaline phosphatase conjugated goat anti-mouse IgG in blocking buffer was added. After a 1-h incubation at 37°C, microplates were washed 5 times and 25 μl of p-nitrophenyl phosphate (pNPP) substrate at 1 mg/ml in carbonate buffer at pH 9.6 was added and further incubated for 60 min at 37°C. Absorbance was read at 405 nm using a SpectraMax plate reader (Molecular Devices, Sunnyvale, CA). About 1100 supernatants from secretor hybridoma clones were screened and 8 monoclonal antibodies (mAbs) were selected for preliminary characterization by Western blot (data not shown). Two mAbs that showed broad reactivity amongst influenza strains were selected for further analysis (F211-11H12 and F211-10A9).

### Antibody production in Chinese Hamster Ovary (CHO) pools

F211-11H12 and F211-10A9 were sequenced by qRT-PCR and plasmids were synthesized (BIO BASIC Inc.). A CHO cell line (CHOBRI55E1-JN, proprietary to NRC) which is inducible with cumate was transfected with linear polyethylenimine (PEIMax, Polysciences) at a DNA/PEI ratio of 1 in 5 [18]. The resulting stable transfected pools produced antibodies named CHO-11H12 and CHO-10A9. Cells were maintained in PowerCHO media (Lonza) supplemented with 50μM MSX, and antibody production was performed in BalanCD CHO growth media (Irvine Scientific) supplemented with 50μM MSX at 32°C.

### Virus and VLP production

Influenza viruses tested in Western blot analysis were produced in eggs and mammalian cells. The egg-based viruses were produced according to standard protocol [16]. Viral production in HEK293 cells was performed in serum-free media (SFM4Transfx-293, Hyclone) at an MOI of 0.01 at 35° C. The supernatant was collected 48hrs post-infection and clarified by centrifugation. The H1N1 A/Puerto Rico/8/34 sample used as a calibrating antigen in the dot blot assay was semi-purified by sucrose-cushion and concentrated 10 times. Viruses production on avian cells were performed by infection of cells grown in suspension with MOIs ranging from of 0.1 to 0.001 and a trypsin concentration of 1 μg/ml. Supernatant were collected at 24 to 48 hours post-infection after a centrifugation (300g, 5 min).

Vaccine-like samples were produced by infecting 13 eggs at 11 days of embryogenesis. Three strains were produced (H1N1/A/California NYMC X-179A, H3N2 A/Texas/50/2012 and B/Massachusetts/2/2012). At 72 hours post-infection, allantoïc fluid was collected and clarified by centrifugation (4000g, 15min). Semi-purification of the bulk was performed by three successive ultracentrifugation (UC) at 164 000g. After 2 hours, pellet of a 20% sucrose cushion was loaded on a sucrose gradient 25–60% and run overnight. A last ultracentrifugation of 2 hours allowed to remove sucrose by pelleting the viruses collected in the fractions. Viral samples were fragmented at 37°C with 1% triton X-100 in PBS and dialysed (Thermo Scientific- 10K MWCO cassettes) in PBS for 2 hours at 4°C. After PBS buffer exchange, a second dialyse was performed overnight. Triton was captured on Biobeads (14.3g/g of Triton X-100) at RT for 40 min. All the steps from fragmentation were performed with smooth agitation. Viruses were inactivated with 0.01% of formaldehyde at 20°C for 72 hours. HA Bulk content was further characterized by SRID assays.

Virus-like particles were produced in plants as described previously [19] and were generously provided by Medicago Inc.

### Western blotting

Samples were heat-denatured at 95°C for 10min in sample buffer (20% SDS), loaded on a 4–15% gel and transferred to a nitrocellulose membrane (LI-COR Biosciences). The membranes were blocked in 5% non-fat dry milk and further incubated with 2μg/ml of primary antibodies overnight at 4°C. Membranes were washed and incubated with infrared-conjugated secondary antibodies and scanned using an Odyssey scanner (LICOR Biosciences, Lincoln, NE). Alternatively, membranes were incubated with HRP-labeled secondary antibodies and revealed by chemiluminescence or on x-ray films.

### Dot blot

A detailed protocol was published for the slot blot assay [20]. In brief, for the dot blot format, samples were denatured in 4M urea for 30min with shaking at room temperature. After serial dilutions, 100ul was loaded into the dot-blot wells in duplicate, along with a standard of known concentration previously quantified by SRID. Samples are then filtered through a nitrocellulose membrane using vacuum on a bio-dot apparatus (BioRad). The membrane is blocked in 5% non-fat dry milk in PBS for 1 hour at room temperature with shaking, followed by overnight incubation with 6μg/ml antibody diluted in Odyssey blocking buffer at 4°C. Infrared-conjugated secondary antibodies were used for detection, along with the Odyssey scanner (LI-COR Biosciences).

### SRID assay

The SRID assay was performed as previously described [14]. Calibration standards and sheep anti-HA serums against H1N1 A/California/7/2009, H3N2 A/Texas/50/2012 and B/Massachusetts/2/2012 were obtained from NIBSC. Samples and standards were treated with 1μg/ml trypsin-TPCK (Affymetrix USB) for 30min at 37°C with constant rocking, followed by 1% Zwittergent 3–14 at room temperature for 30min with rocking. Next, 20μl per well of standards and samples at different dilutions were loaded into the gel and allowed to diffuse overnight in a humidified chamber. The following day, the gels were dried and stained with staining solution (0.25% R250 Coomassie brilliant blue, 50% methanol, 10% acetic acid) for 20min. The gels were rinsed with water and incubated in de-staining solution for 10min (45% methanol, 9% Acetic acid). Pictures of the gels were obtained with a Kodak Imager (Kodak Image Station 440) and the sizes of the immunoprecipitation rings were measured with Image J.

### Competitive ELISA

Viral-like particles (VLPs) produced in plants (*Nicotiana benthamiana)* and expressing HA from H5N1 A/Indonesia/5/2005 (Medicago Inc.) were used to establish a standard curve. The H5 VLPs were previously quantified by SRID and bicinchoninic acid assay (BCA) to determine total protein content. The plate (Costar, high binding) was coated with 4μg/ml H5 VLP overnight. The antibody (800ng/ml) was mixed with different concentrations of H5 VLP sample (denatured in 4M urea for 30minutes). The antibody-antigen complexes are then added to a 96-well plate that was pre-coated with the same H5 VLP. After 1hr incubation at 37°C, the more concentrated the sample, the fewer antibodies will bind to the pre-coated virus. An HRP-labeled secondary is added and incubated for 1hr at 37° C. The signal is revealed with TMB one Component HRP microwell substrate (Bethyl Laboratories).

### Hemagglutination assay

Titration of the influenza viruses by hemagglutination assay was performed in triplicate as previously described [21]. The assay was completed in 96 well v-bottom plates and dilutions were performed with a semi-automated system (Precision XS, Biotek). 100 μl chicken red blood cells (Charles River Laboratories, Canada) at a concentration of 2x10^7^ cells/ml were added to 100ul sample for a final concentration of 1.25%. The plates were left in a covered plastic container at room temperature for 3 hours and scored. The last dilution showing complete hemagglutination was taken as the end point and was expressed as log HA units per ml. For comparison purposes, results were converted to μg HA/ml using equations previously described [22].

### Surface Plasmon Resonance (SPR)

A BIACORE T200 (GE Healthcare) was used to determine the binding affinity (KD) of antibodies F211-10A9, CHO-10A9, F211-11H12 and CHO-11H12 to the peptide immunogen. All SPR experiments were run in PBS containing 0.05% Tween 20 (Teknova Inc.) and 3.4 mM EDTA as a running buffer. An anti-mouse-Fc surface was immobilized to approximately 2200 RUs using the Immobilization Wizard within the T200 control software set to a 2000 RU target. Standard amine coupling of 20μg/mL anti-mouse Fc solution in 10 mM NaOAc pH 4.5 was used. For the binding assay, approximately 350 RUs of antibody to be tested (-10A9 or -11H12) was captured onto a sheep anti-mouse Fc antibody surface by injecting 20μg/mL solution for 300 seconds. This was followed by a single cycle kinetics injection of peptide using a 2-fold dilution series with a top-nominal concentration of 250nM for antibodies -11H2, and 15nM for antibodies -10A9, or PBST running buffer only for referencing. Using a flow rate of 100 μL/min with a 2700 second dissociation, 180 second injections of each peptide or buffer blank were used. The SPR surfaces were regenerated with 10mM glycine pH 1.5 with a contact time of 120 seconds. Sensorgrams were double referenced and data were analyzed within Biacore T200 evaluation software v3.0 (GE Healthcare).

### Computational approach

First, the antibodies were sequenced and modelled with homology modelling techniques [23] as detailed in supplementary material (S1 Appendix). Next, the structures of the various strains of hemagglutinin were either obtained from the Protein Data Bank (PDB) [24] if available, or were generated with homology modelling techniques [25]. In order to denature the hemagglutinin, a monomer is extracted from the homotrimeric structure. The monomer is unfolded following a steered unfolding approach in which the structure is deformed in the torsional space associated with the bounds [26]. After each incremental deformation, the backbone and the side-chains are optimized in order to generate realistic conformations. The process is repeated until the monomer is completely unfolded. As opposed to the conformation of native states, the conformation of an unfolded state is not unique. For this reason, a total of 100 conformations were generated in order to sample the unfolded conformational space. The next step involves the macromolecular docking between the denatured hemagglutinins and the monoclonal antibodies. As the denatured structure is highly flexible, the docking must be performed in two stages (see S1 Appendix). Subsequently, a flexible induced-fit refinement was performed. Finally, we calculated for each complex the contact density map in order to quantitatively measure the interaction between the monoclonal antibodies and the epitope (highly conserved peptide sequence).

## Results

### Detection of multiple influenza strains by immunoblot

**Fig 1:**
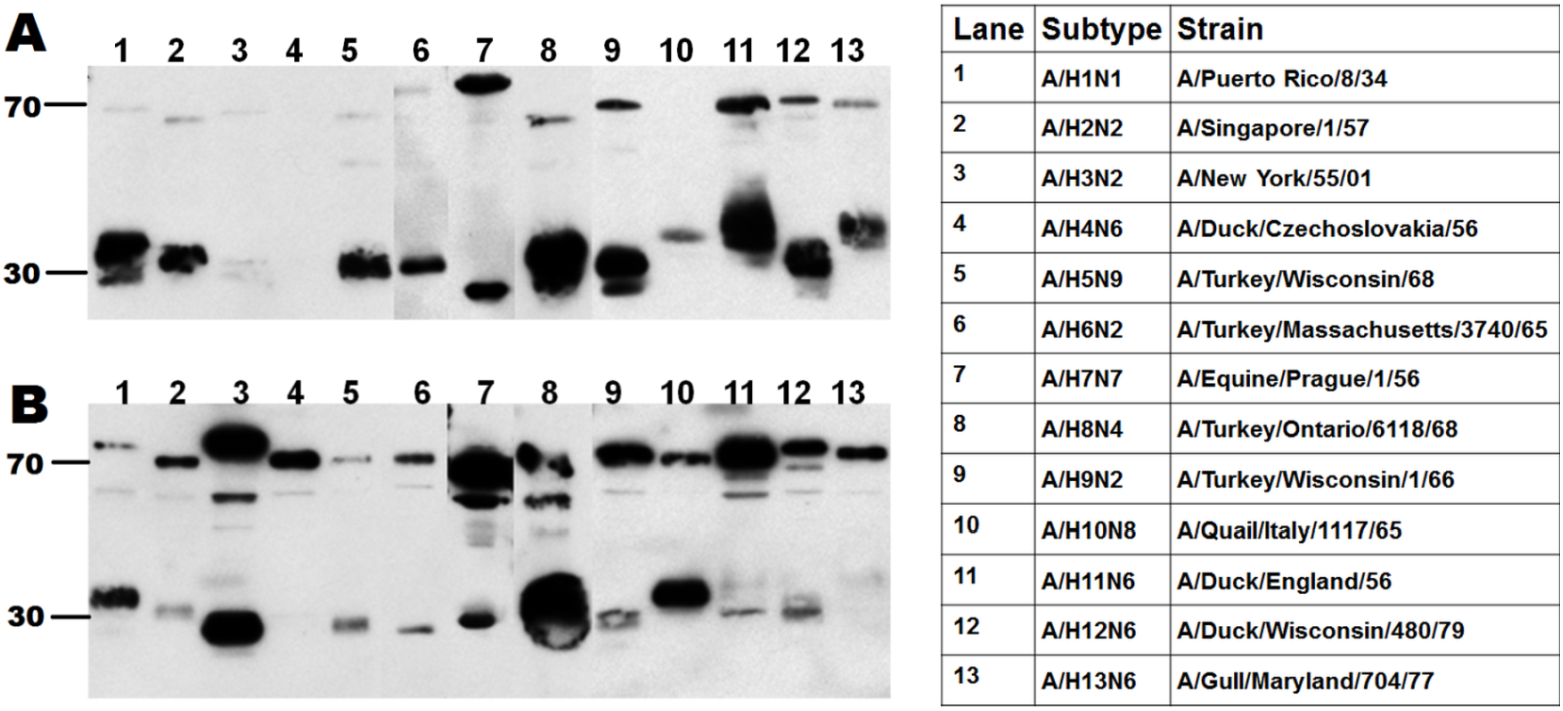
**Binding of mAb F211-11H12 (A) and F211-10A9 (B) to 13 subtypes of influenza.** Molecular markers corresponding to HA2 at 30KDa and HA1 at 70KDa are indicated. Full names of the virus strains corresponding to each lane are shown in the table on the right panel.

Two mouse hybridoma clones were selected and monoclonal antibodies named F211-11H12 and F211-10A9 were generated and purified. The antibodies were first tested by Western blot against thirteen subtypes of influenza viruses (H1 to H13) produced in eggs (Fig 1). All subtypes were recognised by one or both antibodies. Uncropped and unaltered western blots are presented in supplementary material (S1 Fig).

Furthermore, mixing both antibodies resulted in a pan-HA cocktail without cross-inhibition (S2 Fig and S1 Table). Overall, nearly 40 strains of influenza encompassing HA subtypes H1 to H13, as well as B strains from both the Yamagata and Victoria lineage were detected by the pan-HA cocktail by immunoblotting as summarized in Table 1. The panel of strains tested include viruses produced in eggs and mammalian cells, VLPs produced in plants and recombinant HA proteins. It was noticed that the binding affinity for a given strain could vary depending on the production platform. For instance, the signal measured for H1N1/A/California/07/2009 virus preparations varied from low to high when produced in avian cells versus eggs, even though similar concentrations were tested (measured by TCID50). This might be due to different glycosylation patterns [27].

**Table 1:** List of 40 influenza strains and strength of the signal measured when probed with pan-HA mAb cocktail.

Strain	Type of antigen	Production platform	Strength of the signal^1^
H1N1/A/Puerto Rico/8/1934	rHA Virus Virus Virus	Insect cells Eggs MDCK cells HEK293 cells	+++ +++ ++ ++
H1N1/A/California/07/2009	rHA Virus Virus Virus Virus VLP	HEK293 cells Eggs MDCK cells HEK293 cells Avian cells Plant	++ +++ ++ ++ + +++
H1N1/A/Wilson Smith/33	Virus	HEK293 cells	++
H1N1 Avian like Swine	Virus	MDCK cells	+++
H1N2 Reassortant human like A/Scotland/410440/94	Virus	MDCK cells	+++
H2N2 A/Singapore/1/57	Virus	Eggs	++
H3N1/A/Victoria/361/2011	VLP	Plant	+++
H3N2/A/Wisconsin/67/2005	rHA	Insect cells	-
H3N2 A/Aichi/2/1968	Virus	HEK293 cells	+
H3N2 A/Hong Kong/8/1968	Virus	HEK293 cells	+
H3N2/A/Brisbane/10/2007	rHA	Insect cells	+++
H3N2 A/New York/55/01	Virus	Eggs	+++
H3N2 A/Texas/50/2012	Virus	HEK293 cells	+
H3N2A/Panama/2007/99	Virus	Eggs	+++
H4N6 A/Duck/Czechoslovakia/56	Virus	Eggs	++
H5N1/A/Vietnam/1203/2004	rHA	Insect cells	+++
H5N1/A/Indonesia/5/2005	rHA VLP	Insect cells Plant	+++ +++
H5N2 A/Finch/England	Virus	MDCK cells	+++
H5N9 A/Turkey/Wisconsin/68	Virus	Eggs	++
H6N2 A/Turkey/Massachusetts/3740/65	Virus	Eggs	++
H7N7/A/Netherlands/219/2003	rHA	Insect cells	+++
H7N7 A/Equine/Prague/1/56	Virus	Eggs	+++
H7N9 A/Anhui/1/2013	rHA Virus	Insect cells Eggs	++ +
H7N9 A/Shanghai/2/2013	rHA	HEK293 cells	++
H7N9/A/Pigeon/Shanghai/S1069/2013	rHA	HEK293 cells	++
H7N3/A/Turkey/Oregon/71	Virus	Eggs	+++
H7N1 A/Turkey/Italy/977/1999	Virus	MDCK cells	+
H8N4 A/Turkey/Ontario/6118/68	Virus	Eggs	+++
H9N2/A/Hong Kong/1073/1999	rHA	Insect cells	++
H9N2 A/Turkey/Wisconsin/1/66	Virus	Eggs	+++
H10N8 A/Quail/Italy/1117/65	Virus	Eggs	+++
H11N6 A/Duck/England/56	Virus	Eggs	+++
H12N6 A/Duck/Wisconsin/480/79	Virus	Eggs	+++
H12N5 A/Duck/Alberta/60/76	Virus	MDCK cells	+++
H13N6 A/Gull/Maryland/704/77	Virus	Eggs	++
B/Malaysia/2506/2004	Virus	Eggs	+++
B/Brisbane/60/2008	rHA	Insect cells	+++
B/Lee/40	Virus	HEK293 cells	++
B/Massachusetts/2/2012	Virus Virus Virus	Avian cells MDCK cells HEK293 cells	- +++ +++
B/Florida/04/2006	Virus Virus	MDCK cells Eggs	+++ +++

^1^The signal was measured by immunoblotting in Relative Light Units (RLU):–means no signal (<200 RLU), + means low signal (500 to 2000RLU), ++ means medium signal (2000 to 15000 RLU), +++ means strong signal (15000 to 40000 RLU). Negative controls were under 200 RLU.

### Quantification of influenza viruses

**Fig 2:**
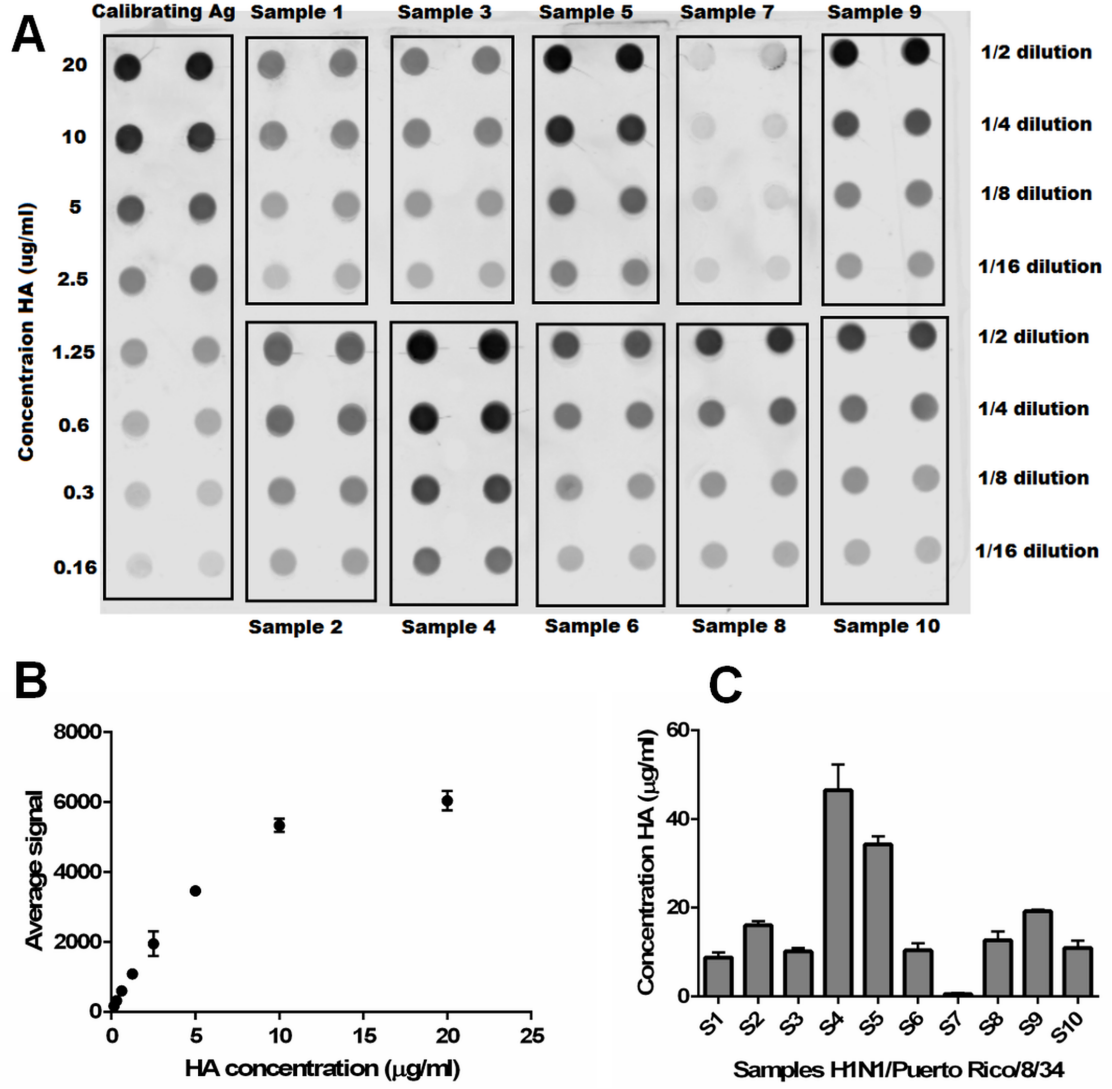
Quantification by dot-blot using the pan-HA cocktail of ten influenza samples (H1N1 A/Puerto Rico/8/34) produced in HEK293 suspension cells. The calibrating antigen (Ag) was loaded in duplicate in concentrations ranging from 160ng/ml to 20μg/ml, and ten samples were loaded in 4 different dilutions as indicated in a representative blot (A). Using the signal measured with the calibrating antigen, a standard curve was generated with an R squared value of 0.9893 in the linear range (B). Using the standard curve, HA concentrations for the ten non-purified samples (S1 to S10) loaded in A were calculated (C).

Influenza virus samples were produced in HEK293 suspension cells under different conditions and quantified by dot blot using a method previously described [16]. A sucrose-cushion semi-purified virus (H1N1 A/Puerto Rico/8/34) was quantified by SRID and used as a calibrating antigen (Fig 2A and 2B). Non-purified samples with concentrations ranging from 0.5 to 46.5μg HA/ml were quantified (Fig 2C) demonstrating the large dynamic range of the method in conjunction with the pan-HA mAbs. Results obtained by dot blot for the most concentrated (S4) and least concentrated (S7) samples were validated by HA assay with values of 4.1 log HA units /ml (corresponds to 41μg HA/ml) and 0 log HA units/ml respectively. These results also show that the method could be applied to in-process samples, a stage at which reference material is needed and often not available yet. Based on the signal presented in Table 1, the strength of the binding of the antibodies to certain strains is low. However, a calibration curve can be generated even for those strains (S3 Fig).

Moreover, samples can be quantified multiple times on different days with coefficient of variation below 5% (Table 2) demonstrating the reproducibility of the dot blot assay in combination with the pan-HA cocktail.

**Table 2:** Concentrations obtained by dot blot on three different runs for two influenza samples (H1N1 A/Puerto Rico/8/34).

	Concentration VLP^1^ (μg HA/ml) [28]	Concentration virus^2^ (μg HA/ml)
**Run 1** **Run 2** **Run 3**	134.7 125.9 132.3	13.3 13.0 12.4
**Average**	131.0	12.9
**Standard deviation**	4.5	0.5
**Coefficient of variation**	3.5	3.5

^1^ VLP: Virus-like particles produced in-house in HEK293 suspension cells

^2^ Virus sample was produced in-house in HEK293 cells and clarified by centrifugation

**Fig 3:**
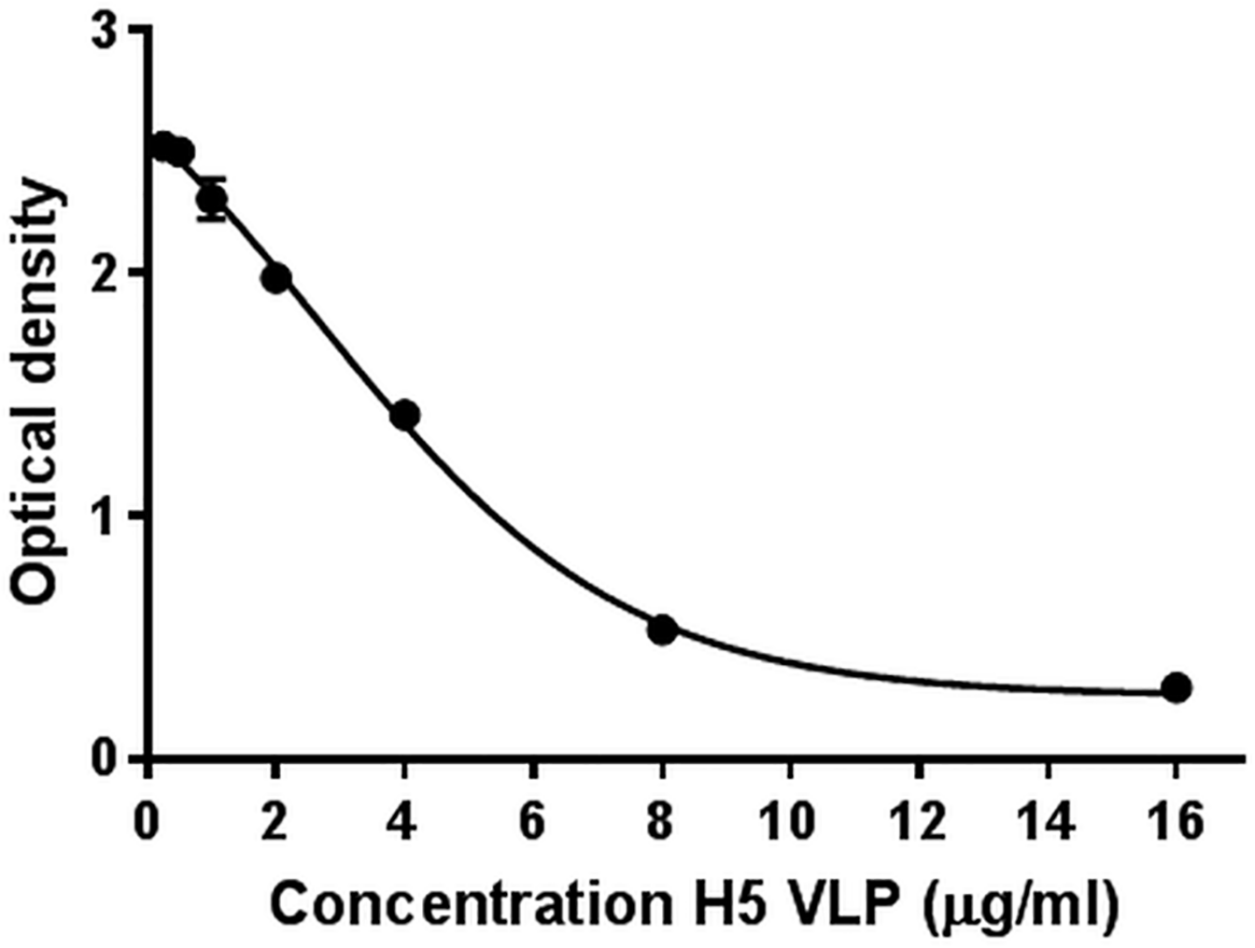
A standard curve was generated with H5 VLP from 0.25 to 16 μg/ml in a competitive ELISA. The antibody at a concentration of 800ng/ml was mixed with different concentrations of H5 VLP and added to a pre-coated plate. The standard curve was obtained by plotting the mean absorbance (y axis) against the VLP concentration (x axis). A four parameter logistic regression was performed to fit the curve.

The ELISA is a widespread immunoassay for protein quantification and is a likely alternative method to the SRID. The competitive ELISA is the most sensitive type of ELISA and requires only one antibody. In a proof of concept, a standard curve was obtained with an H5 VLP based on the sequence of H5N1 A/Indonesia/5/2005 (Fig 3). The lowest concentration of H5 VLP detected was of 250ng/ml.

### Comparison to other quantification methods

**Fig 4:**
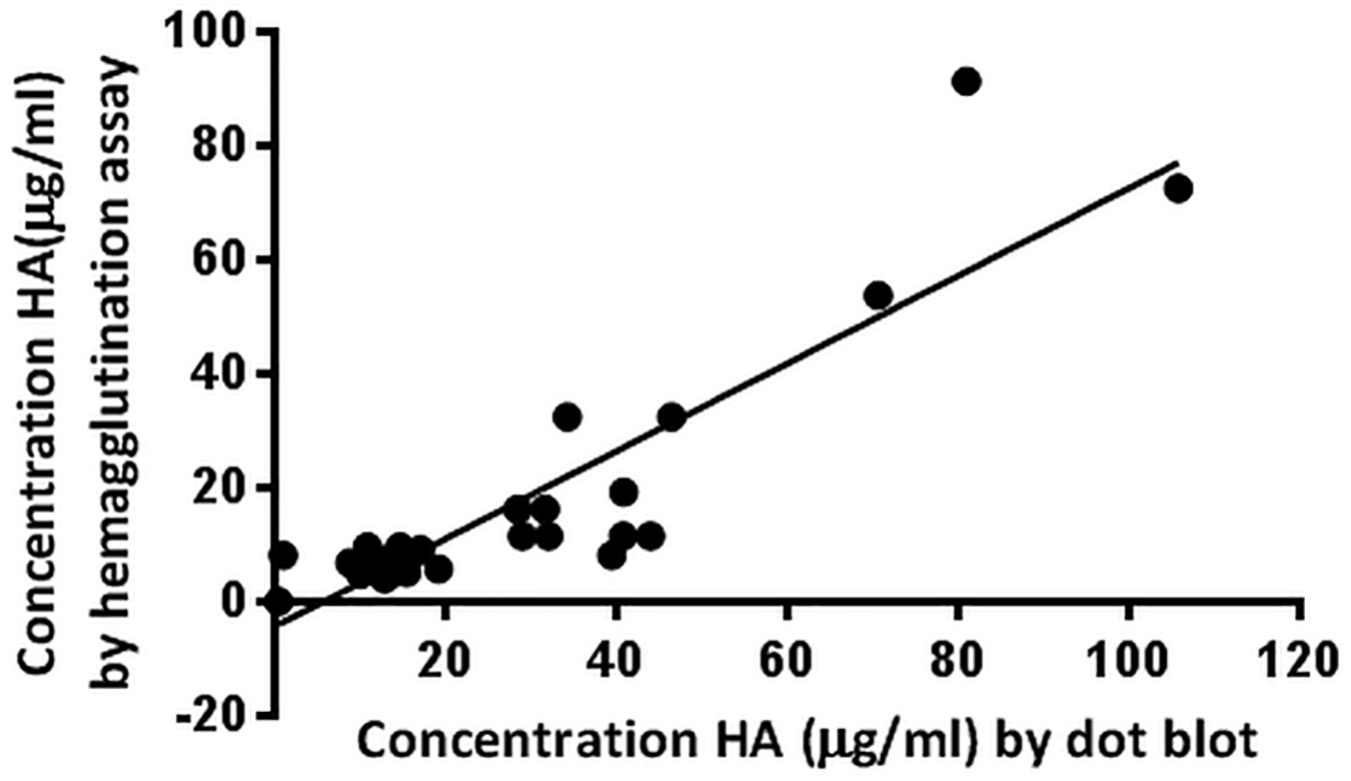
Comparison of HA concentrations obtained by dot blot and hemagglutination assay. Thirty-six samples were produced in HEK293 suspension cells and clarified by centrifugation. Samples were quantified by dot blot using the pan-HA cocktail, and by hemagglutination assay with chicken red blood cells. The R-squared value calculated by linear regression is 0.8015.

Currently, influenza samples are quantified mainly by hemagglutination assay and SRID. Thirty-six in-process samples produced in HEK293 suspension cells were quantified by dot blot and hemagglutination assay (Fig 4), with a coefficient of correlation (R^2^) of 0.80.

The H1N1 A/Puerto Rico/8/34 samples quantified by dot blot and HA assays were not purified and mimic in-process samples. Three additional strains that were produced in eggs, split and deactivated as it is done for vaccines produced by manufacturers were quantified by dot blot and SRID using the same calibrating antigen for each strain (Table 3). The yield of B/Massachusetts/2/2012 was below the threshold of detection of both methods. With the other two strains, the HA concentration measured by dot blot is higher than the one measured by SRID by 13 to 23%.

**Table 3:** HA concentrations of three vaccine-like samples measured by SRID and dot blot.

Strain	Concentration by SRID (μg HA/ml)	Concentration by dot blot (μg HA/ml)	% difference
H1N1 A/California NYMC x-179A	112	128	13
H3N2 A/Texas/50/2012	69	86	23
B/Massachusetts/2/2012	0	0	0

### Hybridoma versus CHO-produced mAb

In order to scale up the production of pan-HA mAb, a different antibody production platform was explored. The variable regions of heavy and light chains of F211-11H12 and F211-10A9 hybridomas were sequenced by qRT-PCR and confirmed by mass spectrometry analysis of the purified mAbs (results not shown). DNA corresponding to the sequences were synthesized, cloned into vectors for full-length mouse IgG expression and transfected in a cumate-inducible CHO cell line using PEI in order to produce stable CHO pools. The two antibodies generated were named CHO-11H12 and CHO-10A9. Their binding affinity was tested by SPR and compared to their hybridomas counterpart; similar KD values were measured for the 4 mAbs, ranging from 3.38E^-10^ to 6.39E^-10^ M (Table 4).

**Table 4:** KD values (M) measured by SPR for pan-HA antibodies produced in mouse hybridomas and CHO pools.

mAb	KD (M)	
	Average	SD
F211-10A9	3.38 E^-10^	7.85E^-11^
CHO-10A9	3.65 E^-10^	4.21E^-11^
F211-11H12	4.65 E^-10^	7.17E^-11^
CHO-11H12	6.39 E^-10^	1.16E^-10^

**Fig 5:**
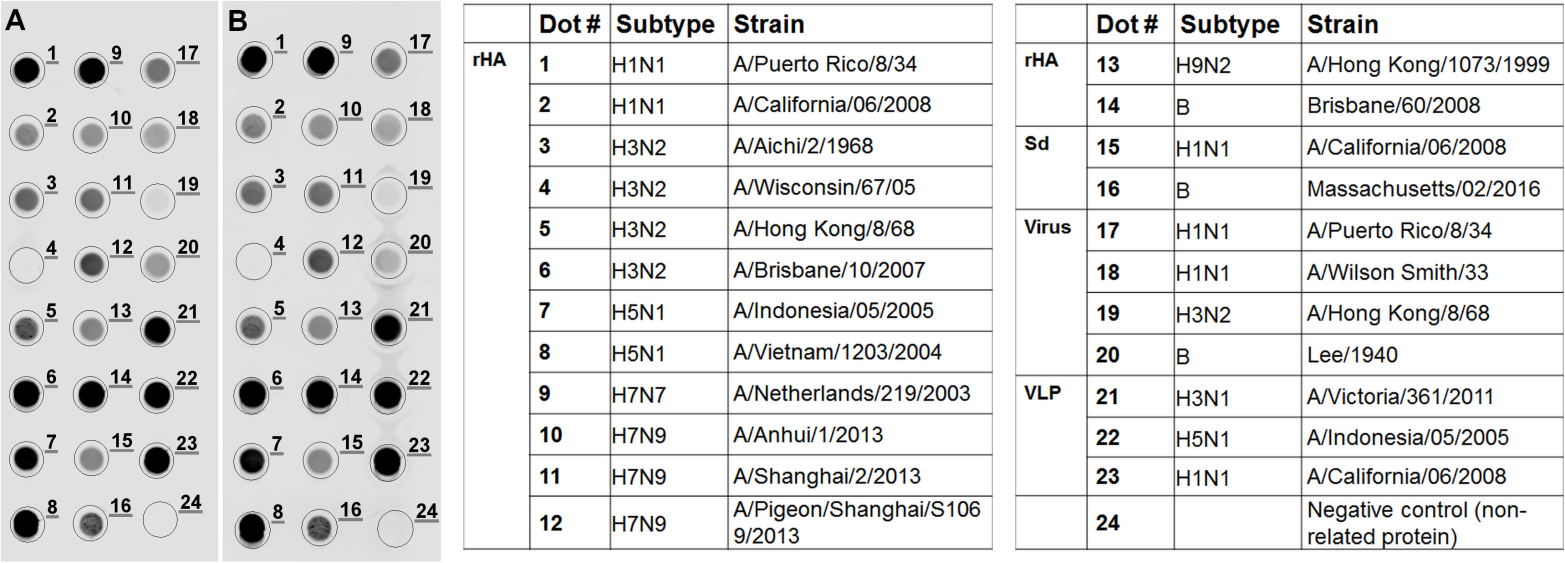
**Dot blot results with a pan-HA cocktail obtained from mouse hybridomas (A) and CHO pools (B).** Each dot represents a different strain as indicated in the table on the right panel. Recombinant proteins and plant VLPs were loaded at a final concentration of 2.5 μg while the viruses and standard antigens were loaded at a concentration of 5 μg. Legend: rHA = recombinant hemagglutinin, Sd = cell-produced calibrating standards (NIBSC), Virus = viruses produced in-house in HEK293 cells, VLP = Viral like particles produced in plants (Medicago).

In addition, the reactivity panel of the CHO-produced mAb cocktail is the same as the one generated by hybridoma-mAbs as demonstrated by dot blot against 18 strains (Fig 5). The intensity of the signal correlates with the binding affinity of the antibody with a given strain. All the strains tested were recognized, except for a recombinant protein from H3N2 A/Wisconsin/67/05 (spot 4) for unknown reason; all other H3N2 strains were efficiently detected. Again, a variation due to the production platform was observed. For instance, the detection of the same strain expressed either as a recombinant protein (spot 5) or a virus (spot 19) differs greatly.

### Computational approach

**Fig 6:**
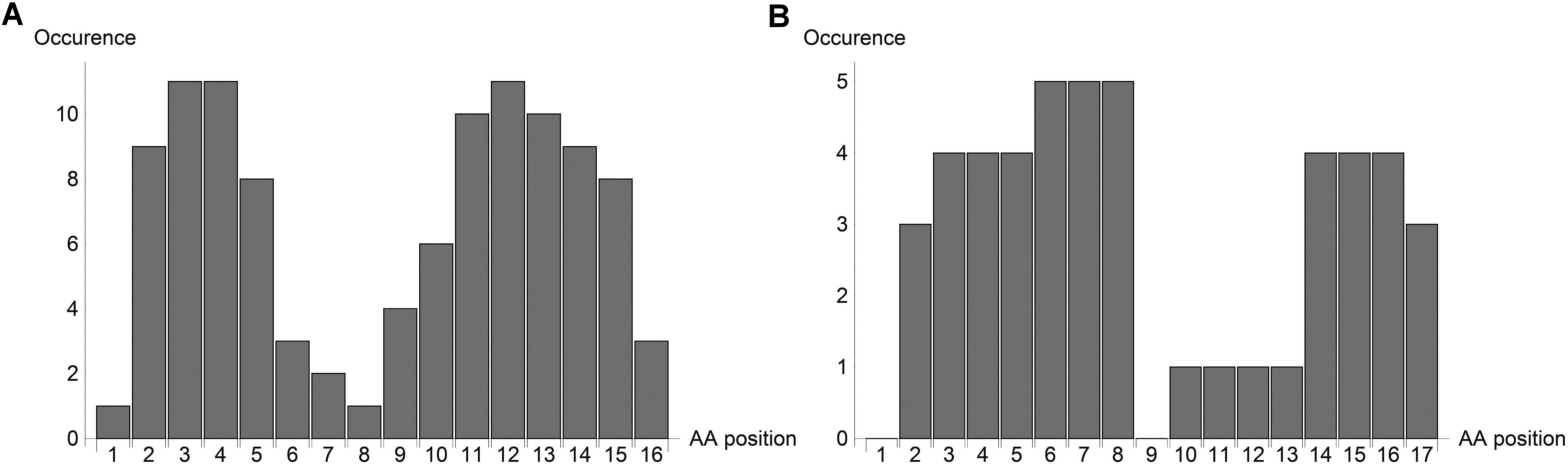
**Probability of interaction between the highly-conserved peptide sequence and mAb 11H12 (A) or mAb 10A9 (B).** Amino Acids 1 to 14 correspond to the highly-conserved peptide used to generate antibodies (GLFGAIAGFIEGGW). Amino acids 15–17 are the amino acids following the end of the highly conserved sequence.

Overall, we have observed that mAb 11H12 preferentially detects HA from influenza subtypes that belong to group 1 (H1, H2, H5, H6, H8, H9, H11, H12, H13, H16, H17 and H18), while mAb 10A9 favours group 2 influenza (H3, H4, H7, H10, H14, and H15). In order to better understand the interaction between the monoclonal antibodies and the highly conserved sequence, an in silico model was built. We calculated, for each complex, the conformational fit between the molecular surfaces associated with the monoclonal antibodies and the molecular surface associated with the highly conserved peptide sequence [29]. The probability of interaction between the antibodies and the amino acids composing the peptide used to raise the antibodies is reported in Fig 6. A clear difference can be observed between the two antibodies; for instance the probability of interaction between 11H12 and amino acids 6 to 8 (IAG) is low, while the opposite is observed for 10A9. Large differences are also observed for amino acids 9–13 (FIEGG). Even though these results were not confirmed experimentally, we can speculate that the different probabilities of interaction may explain the difference in binding affinity of the two antibodies for group 1 and group 2 influenza.

## Discussion

In order to evade the immune system, the influenza virus has evolved by modifying the most exposed portion of the HA molecule through antigenic drift and shift. Thus, different strains emerge and require specific antibodies for detection and quantification purposes. Yet, using bioinformatics tools, a portion of the molecule called the fusion peptide was found to be highly conserved among influenza strains [16]. Our approach to generate pan-HA antibodies is similar to the one employed by Li et al. through the use of a peptide-conjugate derived from the fusion peptide as an immunogen. However, the peptide conjugate used to raise Uni-1 is highly hydrophobic and was not immunogenic in mice, which undermined the generation of hybridomas for monoclonal antibody production [30]. Therefore, modifications were performed on the peptide-conjugate in order to improve its solubility. Immunizing mice instead of rabbits, enabled the generation of monoclonal antibodies with less lot to lot variation and better yield than polyclonal antibodies. The antibodies thus obtained are able to detect all HA subtypes tested as shown by immunoblots (Fig 1). Stability testing of the two mAbs has shown no degradation after 12 months at 4°C in PBS and the binding affinity remains unchanged after 3 freeze-thaw cycles (results not shown) which speaks to the consistency and quality of the pan-HA mAbs.

Western blots were performed in three independent laboratories, using HA recombinant proteins, as well as viruses produced in eggs and in different mammalian cell lines. Antibodies were tested separately in order to better characterize the binding affinity of each mAb. Overall, the results show that mAb 11H12 binds preferentially to influenza group 1 strains, and mAb 10A9 binds preferentially to influenza group 2 strains. Strains belonging to influenza B types presented in Table 1 were recognised by either one of the antibodies. When combining the two mAbs, we obtain a complementary cocktail that allows the detection of all the strains tested. Western blotting can be used as a quantification tool [31]. However, because of the inherent low throughput of the assay, a dot blot assay with 96 wells capacity was optimised. The dot blot assay is easy to implement with minimal costs, and results are reproducible. The main limitation is the need for a calibrating antigen to generate the standard curve. For the H1N1 A/Puerto Rico/8/1934 samples, we have used a sucrose-cushion semi-purified sample that was previously quantified by SRID. For the vaccine-like samples, we used the appropriate calibrating antigens from NIBSC. For new strains, a recombinant protein can be used to generate the standard curve even though the binding affinity might vary depending on the nature of the sample (S4 Fig). Alternatively, a calibrating antigen can be produced and quantified using a method based on SDS-page as recently described [28]. HA concentrations obtained by dot blot were compared to values obtained in the hemagglutination assay and the SRID assay. A correlation of 0.80 was obtained between the dot blot and the hemagglutination assay. The hemagglutination assay relies on the availability of fresh red blood cells, and varies depending on the influenza strain and the type of red blood cells used (chicken, turkey, etc) [32]. It also suffers from a lack of sensitivity which could explain the moderate correlation between the two methods. A difference of 13% to 23% was found between the concentrations measured by dot blot and the ones measured by SRID. This difference could be due to HA conformation. Total HA is measured by dot blot (including monomers, trimers and oligomers) whereas only trimeric HA is measured with the SRID assay [33]. Compared to the SRID, the dot blot assay using pan-HA antibodies circumvents the need for strain-specific antibodies, exhibits less user-dependent variation and offers a higher throughput.

The first generation of antibodies used in this study were produced from mouse hybridomas with a yield of about 100mg/L of culture but higher levels of production are achievable using mammalian expression systems such as CHO cells [34]. The pan-HA antibodies have therefore been sequenced and expressed in CHO cells. Similar binding affinities and specificities were observed between both types of antibodies. As a result, production can easily be scaled up using CHO cells in large bioreactors if needed [35].

Finally, a model was developed to calculate the probability of binding between the two antibodies and the peptide used to raise the antibodies. The different binding profiles might explain the binding affinities exhibited by the two antibodies against the strains tested.

Broadly neutralizing antibodies targeting the stem region of HA have been described and are summarized in recent reviews [15, 36]. Anti-stem antibodies have been generated in mice, humans and from display libraries [36, 37], against either group 1 or group 2 [38–41], or against both groups of influenza [42–44]. Recently, an antibody that can recognize strains from group 1, group 2 as well as strains from the B lineage has been reported [45] but was mostly tested for its neutralizing activity against influenza B viruses for therapeutic applications. The approach explored here is to use anti-stem antibodies to address the enduring quantification issue. The pan-HA antibodies have been tested against a large panel of strains including 13 HA influenza A subtypes as well as B subtypes. In addition, the pan-HA cocktail can detect egg-produced viruses, but also new-generation vaccines such as VLPs. Indeed, the host can also have an impact on detection due to different glycosylation patterns [27].

## Conclusion

Tens of influenza strains produced in eggs, HEK293 cells, MDCK cells, and avian cells were efficiently detected by the pan-HA mAbs. Purified and non-purified viruses and VLPs with concentrations as low as 250ng/mL were quantified by ELISA and dot blot. The monoclonal pan-HA antibodies were generated in mice against a highly conserved sequence found in the fusion peptide. Issues related to heterogeneity such as the ones observed with polyclonal antibodies are thus avoided. More importantly, large quantities could be produced in large-scale bioreactors from CHO-producing cells to respond to industrial demand. This could be critical in case of a pandemic but could also considerably speed up process development. In conclusion, the pan-HA antibodies represent important tools that can assist manufacturers and researchers in the quantification of in-process samples and vaccines.

## Supporting information

S1 Table Signal obtained by dot blot with 6 μg mAb F211-11H12, 6 μg mAb F211-10A9, and 6 μg of both mAb (cocktail). (DOCX)

S1 Appendix Description of the computational approach. (DOCX)

S1 Fig Uncropped and unaltered original blots obtained with viruses produced in eggs and probed with mAb F211-11H12 (Panel A) or mAb F211-10A9 (Panel B). (TIF)

S2 Fig Recombinant HA proteins were detected by mAb F211-11H12 (A), F211-10A9 (B), or a cocktail made of both antibodies (D). An anti-GFP antibody was used as a negative control (C). The influenza strain loaded in each lane is indicated in the table in the bottom panel. Anti-GFP (clone 3E6) negative control mAb was produced and purified in our laboratory and is commercially available (Thermofisher).
(TIF)

S3 Fig Standard curve generated with strains presenting a low signal by dot blot when probed with pan-HA antibodies. A) H3N2 A/Aichi/2/1968 is a non-purified virus produced in-house in HEK293 cells. B) H3N2/A/Texas/50/2012 is a standard reagent produced in HEK293 cells by NIBSC and inactivated with formalin.
(TIF)

S4 Fig Standard curves generated with standards produced using different platforms. A) H1N1 A/Puerto Rico/8/34 virus was produced in HEK293 cells and quantified by SRID. As a comparison, a standard curve obtained using a recombinant protein (Protein Sciences) is shown. B) H1N1 A/California/07/2009 standard from NIBSC (Code 09/174) was produced in MDCK cells and inactivated. A recombinant protein produced in HEK293 cells (Immune Technology) was also used to generate a standard curve using the concentration provided by the manufacturer.
(TIF)
