# Supplementary material for: Generation of monoclonal pan-hemagglutinin antibodies for the quantification of multiple strains of influenza
Source: PLoS One. 2017 Jun 29;12(6):e0180314. doi: 10.1371/journal.pone.0180314 (PMC5491208; doi:10.1371/journal.pone.0180314)
Supplement: S1 Table — (DOCX) [file pone.0180314.s001.docx]

**S1 Table: Signal obtained by dot blot with 6 µg mAb F211-11H12, 6 µg mAb F211-10A9, and 6 µg of both mAb (cocktail).**

|  | **Type of Ag** | **F211-11H12** | **F211-10A9** | **Cocktail** | **% difference*** |
| --- | --- | --- | --- | --- | --- |
| H1N1 A/Puerto Rico/8/34 | rHA | 28617 | 7070 | 31700 | 10 |
| H1N1 A/Puerto Rico/8/34 | Virus | 9965 | 9020 | 11600 | 15 |
| H1N1 A/Wilson Smith/33 | Virus | 9733 | 1870 | 8180 | 17 |
| H1N1 A/California/06/2008 | NIBSC | 26550 | 40300 | 24100 | 10 |
| H3N2 A/Wisconsin67/05 | rHA | 270 | 1540 | 1570 | 2 |
| H3N2 A/Hong Kong/8/68 | rHA | 3560 | 26200 | 26200 | 0 |
| H3N2/A/Brisbane/10/2007 | rHA | 3690 | 53200 | 54200 | 2 |
| H3N2/A/Texas | NIBSC | 1042 | 2220 | 2320 | 4 |
| H5N1 A/Indonesia/05/2005 | rHA | 51488 | 2420 | 40700 | 23 |
| H5N1/A/Vietnam/1203/2004 | rHA | 60912 | 17600 | 43000 | 34 |
| H7N7/A/Netherlands/219/2003 | rHA | 748 | 39000 | 37200 | 5 |
| H9N2/A/Hong Kong/1073/1999 | rHA | 10158 | 7250 | 9860 | 3 |
| B/Brisbane/60/2008 | rHA | 57949 | 44400 | 40300 | 10 |
| B/Massachusetts | NIBSC | 30285 | 5310 | 27100 | 11 |
| B/Lee | Virus | 12570 | 3360 | 9990 | 23 |

* The % difference was calculated by subtracting the value obtained by one of the mAb (the highest binder), from the value obtained by the cocktail, and dividing by the average of the values.
