# Supplementary material for: Generation of monoclonal pan-hemagglutinin antibodies for the quantification of multiple strains of influenza
Source: PLoS One. 2017 Jun 29;12(6):e0180314. doi: 10.1371/journal.pone.0180314 (PMC5491208; doi:10.1371/journal.pone.0180314)
Supplement: S1 Appendix — (DOCX) [file pone.0180314.s002.docx]

**S1 Appendix: Description of the computational approach**

The interaction between the monoclonal antibodies and the various strains of denatured hemagglutinin were assessed using different techniques. We first address the modelling of the sequenced antibodies. Subsequently, the simulation of the denaturation process of the hemagglutinin is explained. This is followed by a description of the macromolecular docking process in between the antibodies and the hemagglutinin as well as an explanation of the extraction of the epitope associated with the highly conserved sequence at molecular level.

*Homology modelling of the monoclonal antibodies:* The sequenced monoclonal antibodies are modelled with homology modelling techniques. This process involves the segmentation of the respective sequences, the finding of homologous templates, the grafting of the segmented sequences on the templates and conformal optimization [Siv09]. In the absence of homologous templates, a hidden Markov process is used in order to generate templates from remote homologues [Kel15]. The objective function associated with conformal optimization is based on a weighted sum of the electrostatics, van der Waals, solvation and residue pair interaction energies, in addition to the rotamers self-energy. The irregular funnel shape of the objective function is taken into account during the optimization process. The backbones are optimized with a Monte Carlo technique. Further, the rotamers are optimized with a simulated annealing technique, which is needed due to the profusion of local minima. The relative orientation of the light and heavy chain is also optimized.

*Denaturation of the hemagglutinin:* Since the Western blot involves denatured proteins, the trimeric hemagglutinins had to be denatured prior to macromolecular docking. The structures of the various strains of hemagglutinin were obtained from the Protein Data Bank (PDB) [Ros14], if available. For the cases where the PDB did not contain the information, the structures were generated with homology modelling techniques [Bia14]. The denatured hemagglutinin provides insight about the membrane fusion process. Indeed, it has been demonstrated “that destabilization of HA at neutral pH, with either heat or the denaturant urea, triggers a conformational change that is biochemically indistinguishable from the change triggered by low pH. In each case, the conformational change is coincident with induction of membrane-fusion activity, providing strong evidence that the fusogenic structure is formed” [Car97]. In order to denature the hemagglutinin, a monomer, which may be either HA or HA2, depending on the Western blot results, is extracted from the homotrimeric structure. Because of the time-scale involved, it is not possible to unfold the monomer with molecular dynamics techniques [Paq15]. Instead, a steered unfolding approach is followed in which the structure is deformed in the torsional space associated with the bounds [Mai05]. After each incremental deformation, the backbone and the side-chains are optimized in order to generate realistic conformations. The process is repeated until the monomer is completely unfolded. As opposed to the conformation of native states, the conformation of an unfolded state is not unique. For this reason, a total of hundred (100) conformations were generated in order to sample the unfolded conformational space.

*Rigid docking in between the monomers and the monoclonal antibodies:* The next step involves the macromolecular docking in between the denatured hemagglutinins and the monoclonal antibodies. As the denatured structure is highly flexible, the docking must be performed in two stages. Firstly, the relative pose of the monoclonal antibodies and the hemagglutinin is estimated with rigid docking techniques. Secondly, the macromolecular complex is refined with flexible docking in order to take the induced fit involved in the docking process into account [Paq13]. Rigid docking is a necessary operation because the energy landscape associated with flexible docking is too intricate to be optimized directly. The choice of the rigid docking technique was dictated from a recent comparison of these techniques against the most recent protein-protein docking benchmark (version 4) [She15] and is also based on the Critical Assessment of PRediction of Interactions (CAPRI) [Pie14]. The rotation space is sampled, while a fast-Fourier transform technique is utilised for the translational space [Pie11]. The objective function involves a weighted sum of the electrostatic interaction, shape complementarity, knowledge-based potential and van der Wall energy. The complex is solvated with implicit solvation.

*Flexible induced-fit refinement:* Flexible induced-fit refinement is important for two reasons: the monomeric hemagglutinin is highly flexible while the monoclonal antibodies may experience significant conformational changes. The deformation of the backbone is parameterized in terms of normal modes [Mas09]. It has been shown that these modes are correlated with the repulsive van der Waals interaction. Both the modes and the side-chains are optimized with Monte Carlo techniques. The objective function, which is specific to antibodies, involved the electrostatic, van der Waals and paring interaction, as well as solvation. For each complex, the best hundred (100) poses, as obtained previously with rigid docking, are optimized with flexible docking. The latter results are then clustered and the archetype of the best cluster is chosen as the most representative macromolecular complex.

*Extraction of the epitope at atomic level:* In order to quantitatively measure the interaction in between the monoclonal antibodies and the highly conserved sequence, we calculated, for each complex, the contact density map [Che15]. This map measures the conformational fit in between the molecular surfaces associated with the monoclonal antibodies and the molecular surface associated with the various strains of hemagglutinin. The abscissa corresponds to the amino acids of the highly conserved sequence while the ordinate measures the conformational fit. The latter is zero when the conformational fit is perfect, negative when there are some gaps or cavities and positive when there are some clashes. The colour code corresponds to the probability, for a given atom, first, to belong to a specific amino acid and, second, to have a certain level of conformational fit for a given hemagglutinin strain and monoclonal antibody. We use a thermal scale in which the blue colour is associated with lower probability while the red colour is associated with higher probability. The results for the interaction of various strains of influenza with mAb F211-9D1-2, mAb F211-10A9-2 and mAb F211-11H12-2 are shown in Figures 1 and 2 (HA2 – mAb F211-9D1-2), 3 (HA – mAb F211-10A9-2) and 4 to 6 (HA2 – mAb F211-11H12-2), respectively. In this section, we shall only discuss Figure 7 as comparable results are obtained for the above-mentioned complexes. This figure is particularly interesting as it is associated with a blind test for which the results were predicted in silico, prior to any form of experimentation. Figure 7 shows the contact density maps for the complexes A/Shanghai/2/2013 – mAb F211-10A9-2 and A/Shanghai/2/2013 – mAb F211-11H12-2 respectively. As it may be observed from the contact density maps, the interaction with mAb F211-10A9-2 involves much more amino acids and atoms than the interaction with mAb F211-11H12-2. Consequently, the binding with the former should be much stronger than the binding with the latter. This is exactly what was observed experimentally. Consequently, we may conclude that it is possible to assess the nature and the strength of the binding in between the highly conserved sequence (the epitope) and the pan-hemagglutinin antibodies.
