# Supplementary figures and images for: Generation of monoclonal pan-hemagglutinin antibodies for the quantification of multiple strains of influenza
Source: PLoS One. 2017 Jun 29;12(6):e0180314. doi: 10.1371/journal.pone.0180314 (PMC5491208; doi:10.1371/journal.pone.0180314)

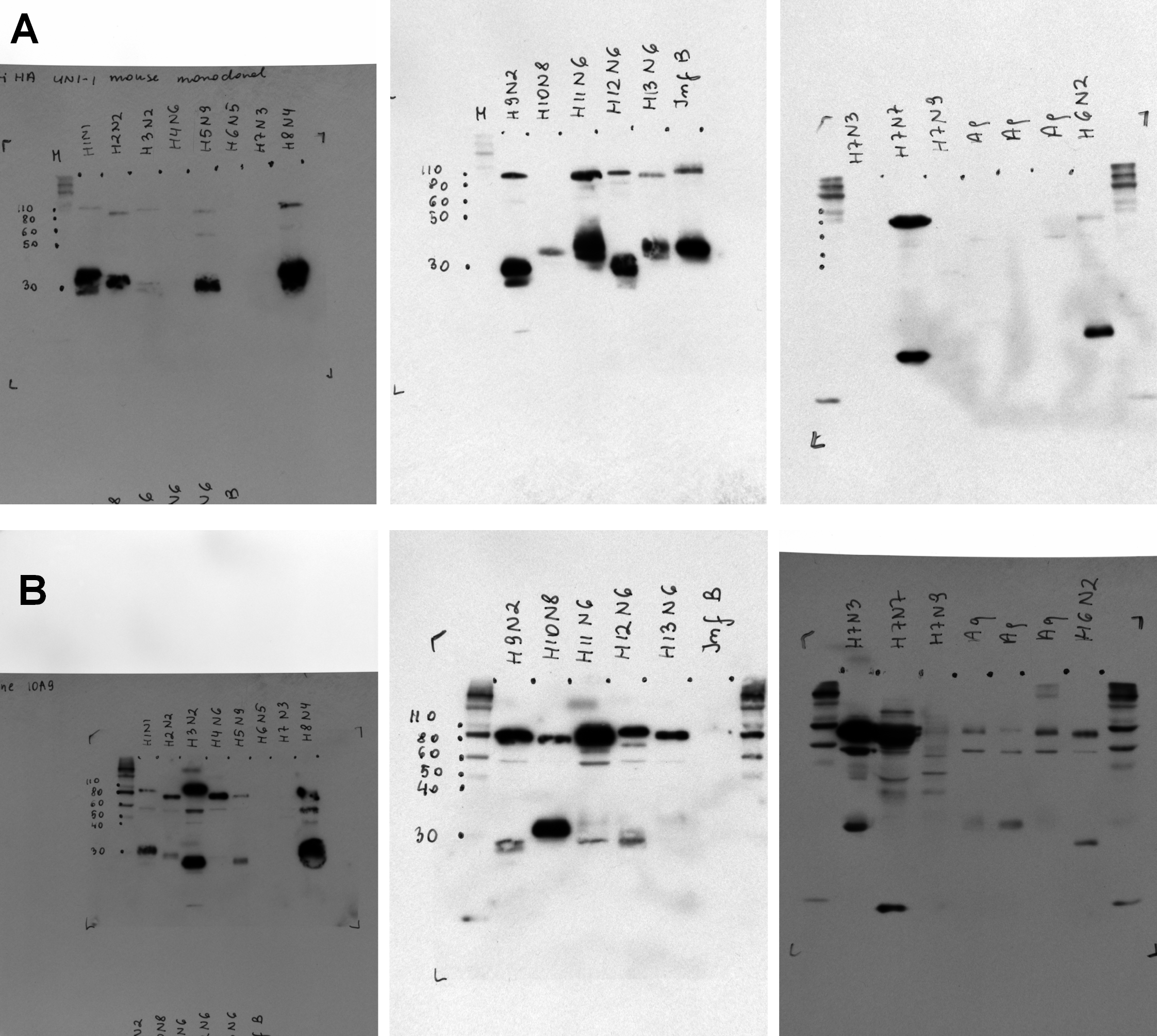

Supplement: S1 Fig — (TIF) [file pone.0180314.s003.tif]

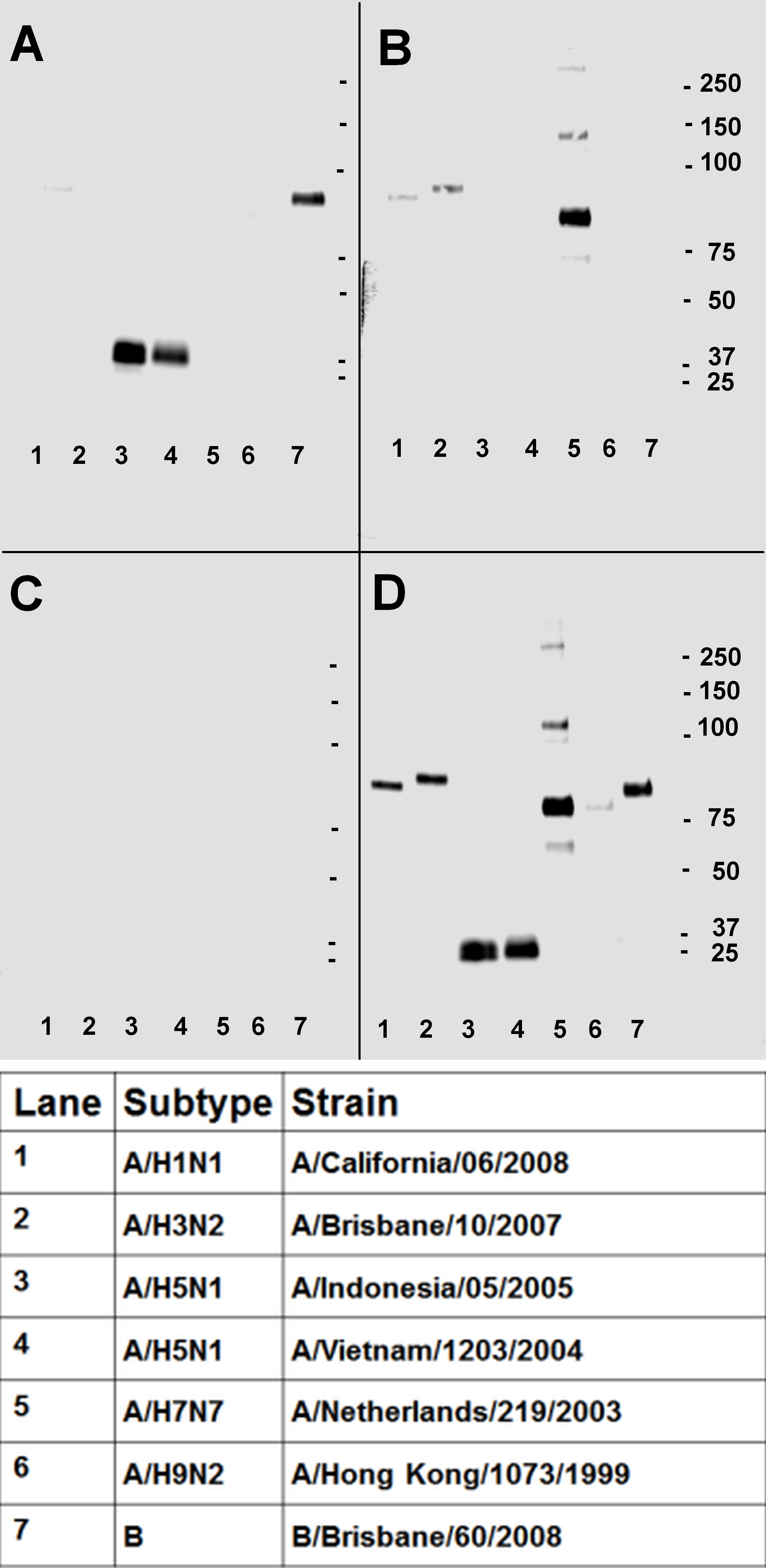

Supplement: S2 Fig — Recombinant HA proteins were detected by mAb F211-11H12 (A), F211-10A9 (B), or a cocktail made of both antibodies (D). An anti-GFP antibody was used as a negative control (C). The influenza strain loaded in each lane is indicated in the table in the bottom panel. Anti-GFP (clone 3E6) negative control mAb was produced and purified in our laboratory and is commercially available (Thermofisher). (TIF) [file pone.0180314.s004.tif]

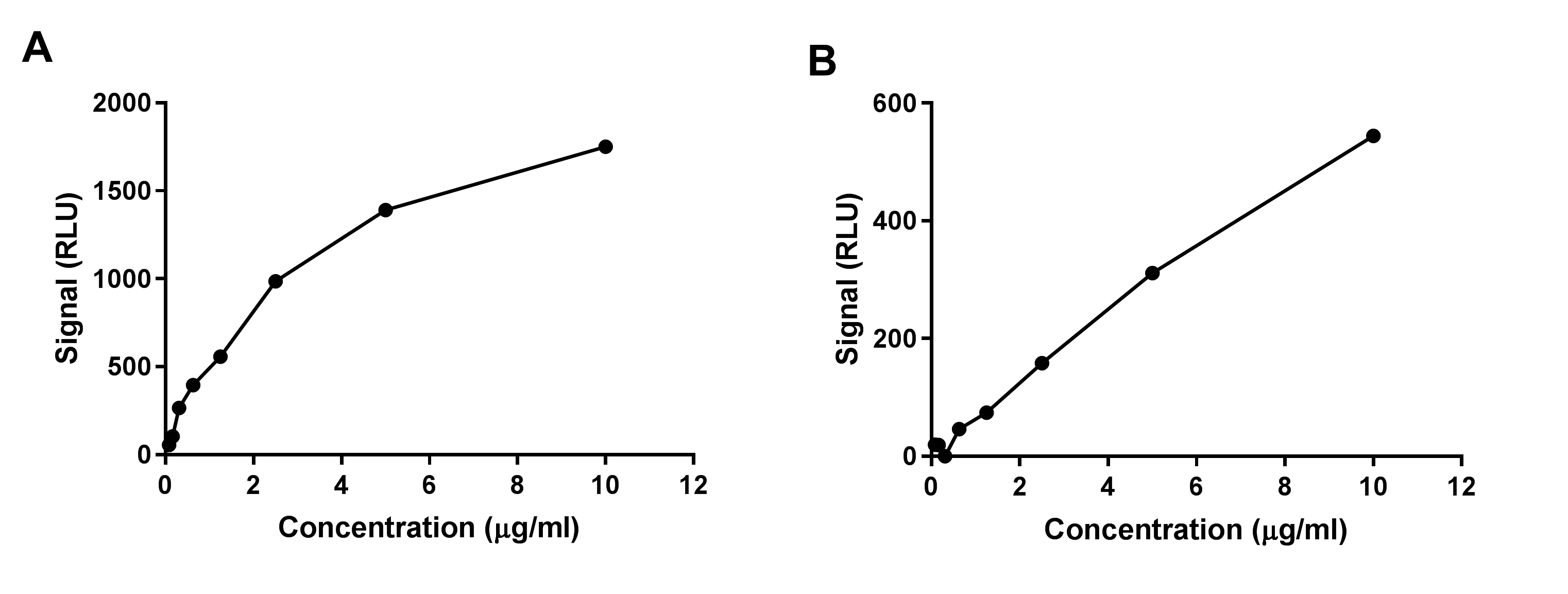

Supplement: S3 Fig — A) H3N2 A/Aichi/2/1968 is a non-purified virus produced in-house in HEK293 cells. B) H3N2/A/Texas/50/2012 is a standard reagent produced in HEK293 cells by NIBSC and inactivated with formalin. (TIF) [file pone.0180314.s005.tif]

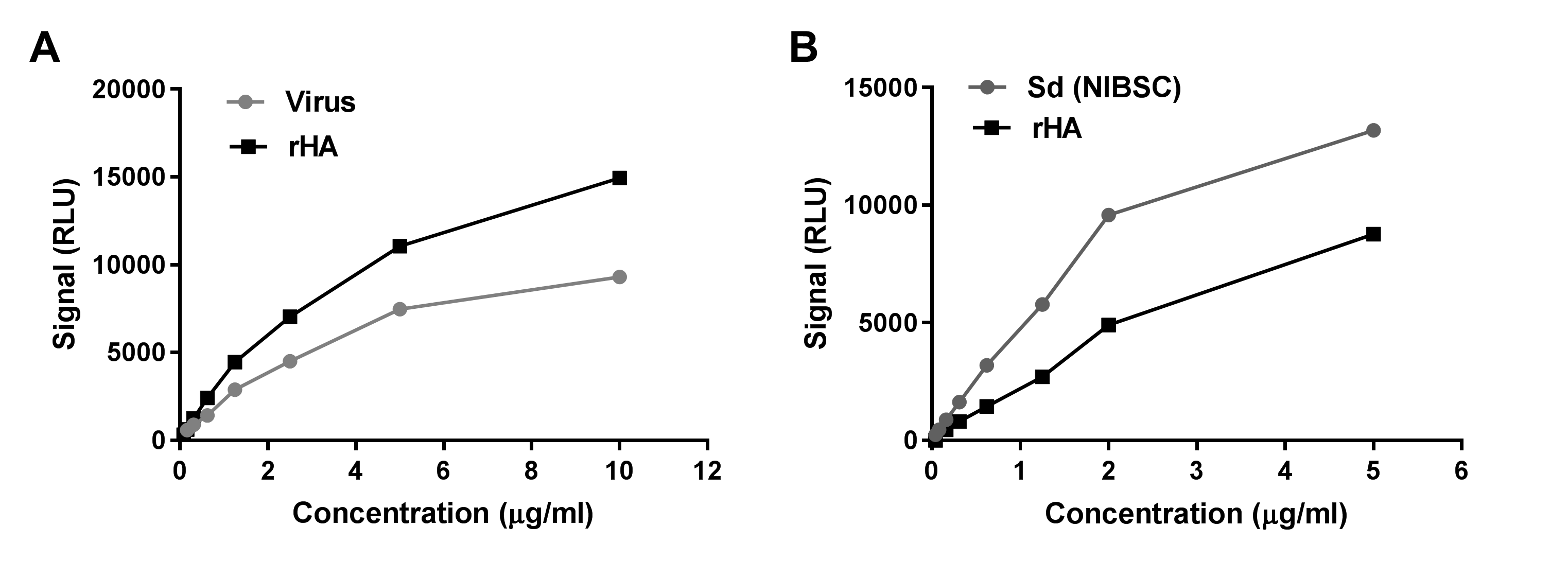

Supplement: S4 Fig — A) H1N1 A/Puerto Rico/8/34 virus was produced in HEK293 cells and quantified by SRID. As a comparison, a standard curve obtained using a recombinant protein (Protein Sciences) is shown. B) H1N1 A/California/07/2009 standard from NIBSC (Code 09/174) was produced in MDCK cells and inactivated. A recombinant protein produced in HEK293 cells (Immune Technology) was also used to generate a standard curve using the concentration provided by the manufacturer. (TIF) [file pone.0180314.s006.tif]
